# A new species of *Megoura* (Hemiptera, Aphididae) from Japan

**DOI:** 10.3897/zookeys.417.7167

**Published:** 2014-06-18

**Authors:** Wonhoon Lee, Takashi Kanbe, Shin–ichi Akimoto

**Affiliations:** 1 Laboratory of Systematic Entomology, Graduate School of Agriculture, Hokkaido University, Kita Ku, Sapporo, 060–8589, Japan

**Keywords:** Macrosiphini, *Megoura*, *Lathyrus japonicus* subsp. *japonicus*, new species

## Abstract

A new species of the genus *Megoura*, *M. lathyricola* **sp. n.**, was collected from *Lathyrus japonicus* subsp. *japonicus* (Leguminosae) in seashore areas of northern and southern Japan. This species is described and illustrated, and a revised key to the identification of the world species of *Megoura* is presented.

## Introduction

The genus *Megoura* Buckton 1876, nested within the tribe Macrosiphini of the subfamily Aphidinae (Remaudière and Remaudière 1997), comprises eight valid species described from the Palearctic, Oriental, and Australian regions (Miyazaki 1971; Heie 1995; Blackman and Eastop 2006; Lee et al. 2002; Eastop 2011). The species are *Megoura lespedezae* (Essig and Kuwana 1918), *Megoura crassicauda* Mordvilko, 1919, *Megoura brevipilosa* Miyazaki, 1971, *Megoura nigra* Lee, 2002 from East Asia, *Megoura dooarsis* (Ghosh & Raychaudhuri, 1969) from the Indian subregion, *Megoura viciae* Buckton, 1876, *Megoura litoralis* Müller, 1952 from Europe, Central Asia and the Middle East, and *Megoura stufkensi* Eastop, 2011 from New Zealand. This genus is characterized by having swollen siphunculi and an association with several genera of Leguminosae (Blackman and Eastop 2006).

Until now, three species of the genus *Megoura* have been reported from Japan; *Megoura crassicauda*, *Megoura lespedezae*, and *Megoura brevipilosa*. Recently, we collected a macrosiphine aphid species from the leguminous plant *Lathyrus japonicus* subsp. *japonicus*, in Hokkaido (northern Japan) and Nagasaki Prefecture (southern Japan). This species has morphological characters in common with the genus *Megoura*, such as swollen siphunculi, smooth head, and antenna approximately as long as body (Miyazaki 1971; Heie 1995). Morphological identification keys and original descriptions of *Megoura* spp. (Miyazaki 1971; Heie 1995; Lee et al. 2002; Eastop 2011) suggest that this species is referable to *Megoura crassicauda* in Japan. However, the apterous viviparous female of this species is distinguished from *Megoura crassicauda* by antennal segment III with 40–59 secondary rhinaria, abdominal tergite III with 11–13 setae, pale tibia (except distal 1/9 dark), and pale yellow cauda.

The present paper describes this new species, and provides a revised key to species of *Megoura* of the world.

## Materials and methods

Aphid samples for this study were collected in 2012 on *Lathyrus japonicus* subsp. *japonicus* in Japan. Each sample of aphid colonies was preserved in 80% alcohol, and mounted specimens were prepared in Canada balsam, following methods by Blackman and Eastop (2000). Illustrations for each species were taken by digital camera, Carl Zeiss, AxioCam MRc5 attached on the microscope, Carl Zeiss Microimaging GmbH 37801, Gottingen, Germany. Measurements for each specimen are taken from the digital images by the software, Axio Vision Re. 4.8.

Abbreviations used for descriptions and table are as follows: al. – alate viviparous female, alata; apt. – apterous viviparous female, aptera; Ant. – antennae; Ant.I, Ant.II, Ant.III, Ant.IV, Ant.V, Ant.VI, and Ant.VIb – antennal segments I, II, III, IV, V, VI, and base of VI, respectively; BDAnt.III – basal diameter of antennal segment III; BL – length of body; GP – genital plate; 2HT – second segment of hind tarsus; PT – processus terminalis; SIPH – siphunculi; URS – ultimate rostral segment (segment IV + V).

## Taxonomy

### Megoura lathyricola

Taxon classificationAnimaliaHemipteraAphididae

Lee & Akimoto sp. n.

http://zoobank.org/2B8624A7-184C-438C-B439-779B72966F5B

**Figure 1.**
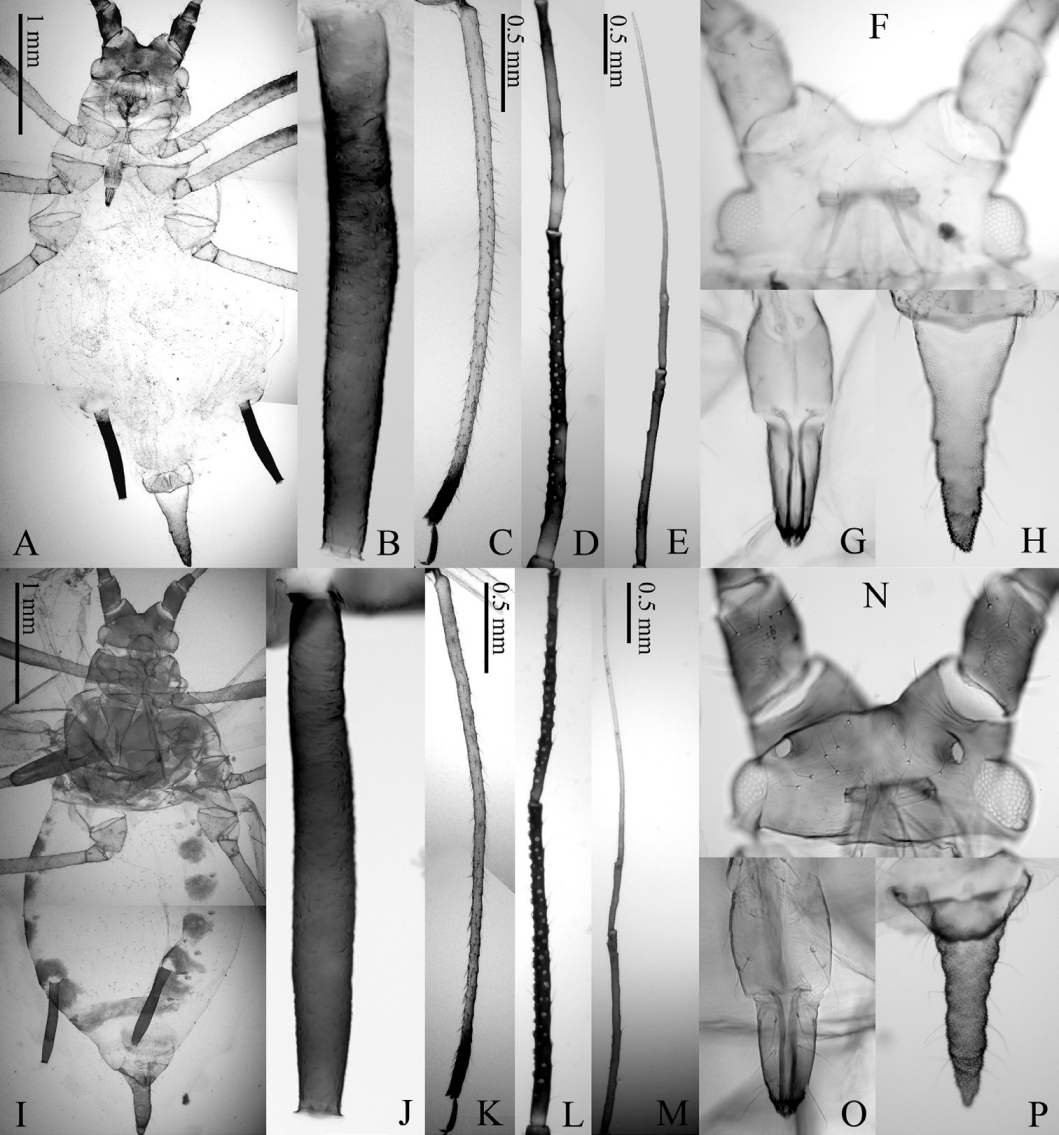
Apterous viviparous female (**A–H**) and Alate viviparous female (**I–P**) of *Megoura lathyricola* sp. n.: **A** whole body of apterous vivipara **B** siphunculus **C** hind tibia and tarsus **D** antennal segments III–IV **E** antennal segments V–VI **F** head focused on dorsum **G** ultimate rostral segment **H** cauda **I** whole body of alate vivipara **J** siphunculus **K** hind tibia and tarsus **L** antennal segments III–IV **M** antennal segments V–VI **N** head focused on dorsum **O** ultimate rostral segment **P** cauda.

Figs 1 , 2 , Table 1

#### Holotype.

Apterous viviparous female, Coll#.120605WH26/ap.1, Muroran, Hokkaido, Japan, 05.vi.2012, leg. Wonhoon Lee, on *Lathyrus japonicus* subsp. *japonicus*.

#### Paratypes.

9 apterous viviparous females and 2 alate viviparous females, same data as for holotype; 5 apterous viviparous females, Coll#.120309WH16, Nagasaki, Nagasaki prefecture, Japan, 09.iii.2012, leg. Wonhoon Lee, on *Lathyrus japonicus* subsp. *japonicus.*

The type specimens, including holotype and paratypes, are deposited in the Laboratory of Systematic Entomology, Graduate School of Agriculture, Hokkaido University, Japan.

#### Etymology.

The specific epithet is composed of the root of the plant genus (lathyr) and the lexeme “col” that, in this case, means ‘living on’.

#### Apterous viviparous female.

(Figs 1A–H, 2A) *Color alive*: Head yellowish brown with antennae dark brown, thorax and abdomen pale green. Legs pale yellowish brown except distal 1/9 of tibiae including tarsi dark brown. SIPH dark brown. Cauda pale. *Color of macerated specimens*: Head yellowish brown and antennae dark brown. Rostrum pale, except the tip of URS dark brown. Thorax yellowish brown and abdomen entirely pale. Legs pale except distal 1/9 of tibiae and tarsi dark brown. SIPH dark brown and cauda pale except extreme end. *Morphology*: Body spindle shaped. Head: smooth on ventrum and dorsum including three pairs of acuminate setae. Antennal tubercle well developed with 3–5 setae on both side, frons U–shaped with four setae on vertex, including weakly developed median tubercle. Ant.I smooth; Ant.II granulate; Ant.III weakly imbricate with short setae, bearing 40–59 secondary rhinaria irregularly spaced; Ant.IV imbricate with 12–18 setae with no secondary rhinaria; Ant.V imbricate with 8–13 setae, primary rhinarium ciliate, longest diameter of which is shorter (0.63–0.83 times) than middle width; Ant.VI imbricate with 3–7 short setae on Ant.VIb. Rostrum attaining posterior margin of mesocoxa; mandibular laminae with 4–6 setae on each side; URS longest seta 0.78–1.00 times as long as apical primary ones. Thorax: pronotum smooth with two spinal setae and one marginal seta on anterior margin. Hind coxa weakly spinulose with 6–7 acuminate setae; hind trochanter wide at base, 1.63–1.65 times as long as apical width, bearing three setae; hind femur smooth on basal 1/17, spinulose on apical 16/17 ventrally, bearing short setae, longest seta 0.43–0.48 times as long as basal width of segment; hind tibia smooth, longest seta 0.80–1.07 times as long as middle width of segment; first segment of each tarsus smooth with three setae at apex; 2HT imbricate with 10–11 setae. Abdomen: dorsum smooth, membranous with 11–13 setae on tergite III, 0.37–0.47 times as long as basal width of hind femur. SIPH swollen, weakly spinulose except smooth base, irregularly reticulated on distal end, apex flanged. Cauda elongate, triangular, ventral spinules strong and dense, in groups of one or two; dorsal ornamentation composed of ribbed imbrication.

**Figirue 2.**
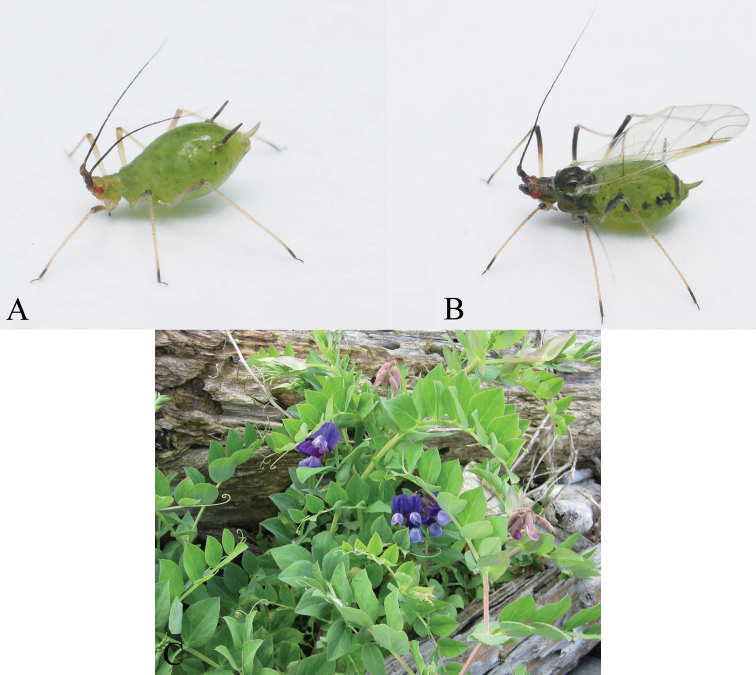
Photographs of *Megoura lathyricola* sp. n. and host plant: **A** apterous viviparous female **B** alate viviparous female **C** *Lathyrus japonicus* subsp. *japonicus*.

#### Alate viviparous female.

(Figs 1I–P, 2B) *Color alive*: Head, antennae and thorax black, abdomen pale green. Abdomen with black patches on tergites III–VI. Legs pale yellowish brown except distal 1/2 of femora and distal 1/9 of tibiae including tarsi dark brown. SIPH dark brown. Cauda pale. *Color of macerated specimens*: Head, antenna, and thorax dark brown. Legs pale except dark distal 1/2 of femora, 1/9 of tibiae and tarsi. Abdomen with marginal sclerites on II–VI segments. SIPH and Cauda dusky. Wings pale with veins bordered by narrow dark pigmentation. *Morphology*: Antennae with 60–62 secondary rhinaria irregularly scattered on whole of Ant.III, and 20–32 secondary rhinaria scattered on whole of Ant.IV. Cauda triangular, pointed at apex. SIPH strongly imbricate. Otherwise like apterous viviparous female.

#### Distribution and host-plant.

So far collected and observed only on *Lathyrus japonicus* subsp. *japonicus* in Muroran, Hokkaido and Nagasaki, Japan. This plant is distributed only in seashore areas.

#### Biology.

This species seems to be rare. Colonies were observed on stem and young leaves of host plants. In nature it appears to be specific to *Lathyrus japonicus*. However a clonal lineage from Nagasaki was readily reared in the laboratory of Systematic Entomology, Hokkaido University under long day conditions by using broad bean seedlings as a host. When the clone was reared at 15 °C and 8L16D, it produced viviparae but no sexual morphs, suggesting that the Nagasaki population of this species is anholocyclic.

**Table 1.** Biometric data of *Megoura lathyricola* sp. n.

	Part	Apterous vivipara (n=10)	Alate vivipara (n=2)
		Range(Mean)	Range(Mean)
Length (mm)	BL	4.06–4.47(4.30)	4.13–4.86(4.49)
	Whole antennae	4.11–4.75(4.40)	4.62–4.91(4.76)
	Ant.I	0.20–0.24(0.22)	0.22–0.26(0.24)
	Ant.II	0.11–0.16(0.14)	0.13–0.16(0.14)
	Ant.III	1.09–1.26(1.18)	1.22–1.28(1.25)
	Ant.IV	0.65–0.90(0.80)	0.91–0.96(0.93)
	Ant.V	0.70–0.78(0.74)	0.75–0.82(0.75)
	Ant.VIb	0.22–0.30(0.27)	0.27–0.35(0.31)
	PT	1.01–1.12(1.04)	1.03–1.20(1.11)
	URS	0.13–0.15(0.14)	0.13–0.14(0.14)
	Hind femur	1.38–1.58(1.48)	1.53–1.59(1.56)
	Hind tibia	2.50–2.78(2.60)	2.72–2.81(2.78)
	2HT	0.16–0.21(0.19)	0.19–0.20(0.19)
	SIPH	0.60–0.73(0.68)	0.58–0.66(0.62)
	Cauda	0.56–0.66(0.62)	0.54–0.57(0.55)
	Setae on Ant.III	0.03–0.06(0.05)	0.04–0.05(0.05)
No. of hairs on	Mandibular lamina.	4–6(5)	5–6(6)
	Ant.I	9–18(13)	10–12(11)
	Ant.II	5–7(6)	5–5(5)
	Ant.III	22–33(26)	20–25(22)
	URS (subsidiary)	6–6(6)	6–6(6)
	Abdominal tergite VI between SIPH	5–7(6)	6–6(6)
	Abdominal tergite VIII	6–7(6)	6–6(6)
	Median of genital plate	2–2(2)	2–2(2)
	Posterior margin of genital plate	17–21(19)	18–18(18)
	Cauda	10–13(12)	13–14(14)
No. of Rhinaria on	Ant.III	40–59(48)	60–62(61)
	Ant.IV	0	20–32(25)
Ratio (times)	Whole Antennae / BL	0.96–1.06(1.02)	1.00–1.13(1.06)
	PT / Ant.VIb	3.50–4.75(3.85)	3.41–3.80(3.58)
	PT / Ant.III	0.83–0.95(0.88)	0.84–0.95(0.89)
	URS / 2HT	0.67–0.82(0.75)	0.69–0.76(0.73)
	URS / Ant.VIb	0.43–0.64(0.52)	0.39–0.53(0.46)
	SIPH / BL	0.14–0.16(0.16)	0.12–0.16(0.14)
	SIPH / Ant.III	0.47–0.64(0.58)	0.46–0.54(0.50)
	SIPH / Hind femur	0.39–0.50(0.46)	0.37–0.43(0.40)
	SIPH / Cauda	1.00–1.16(1.09)	1.08–1.15(1.12)
	Cauda / Width of cauda	1.92–3.03(2.49)	2.10–2.23(2.17)

### Key to species of the genus *Megoura* of the world (based on the apterous viviparous female)

**Table:** 

1	Median cephalic frontal tubercle well developed, and Ant.III with no secondary rhinaria. Cauda with 5–7 hairs. On the genus *Carmichaelia*. In New Zealand	*Megoura stufkensi*
–	No median cephalic frontal tubercles, and Ant.III with 0–64 secondary rhinaria. Cauda with more than 10 hairs	2
2	Cauda dark brown or black. On the genus *Vicia* or *Lathyrus*	3
–	Cauda pale yellow. Not on the genus *Vicia*	5
3	Body totally dark brown or black in life. Tibia pale yellow except apical 1/5. URS 0.88–1.00 times as long as 2HT. Ant.I, mandibular lamina, abdominal tergite III, and GP with 13–20, 6–8, 16–21, and 24–33 hairs, respectively. Ant.III with 28–67 secondary rhinaria. On the genus *Vicia* (Leguminosae). In Korea	*Megoura nigra*
–	Body green except antenna, legs, SIPH, and cauda black in life. Tibia black or dark brown. URS 0.63–0.87 times as long as 2HT. Ant.I, mandibular lamina, abdominal tergite III, and GP with 8–15, 4–5, 15–18, and 14–23 respectively. Ant.III with 5–42 secondary rhinaria	4
4	Ant.III with more than 20 secondary rhinaria which are scattered irregularly over two–thirds of, or throughout the segment. SIPH as long as or longer than cauda. On the genera *Vicia*, *Lathyrus*, *Amphicarpaea*, and *Pisum* (Leguminosae). In Japan, China, India, Korea, Russia (Siberia), and Taiwan	*Megoura crassicauda*
–	Ant.III with less than 20 secondary rhinaria which are situated on basal half or two–thirds, in a line. SIPH shorter than cauda. On the genera *Vicia* and *Lathyrus* (Leguminosae). In Europe, Central Asia, Middle East, and Ethiopia	*Megoura viciae*
5	All legs pale, except that apices of tibiae and tarsi are pale brown	6
–	Legs dark brown, or at least fuscous on distal half of femora and tibiae	7
6	SIPH 0.67–0.95 times as long as cauda. Ant.III with 6–21 secondary rhinaria. PT 2.70–3.70 times as long as Ant.VIb. On the genus *Lathyrus* (Leguminosae). In Denmark, Finland, Germany, Norway, Poland, and Sweden	*Megoura litoralis*
–	SIPH 1.00–1.16 times as long as cauda. Ant.III with 40–59 secondary rhinaria, PT 3.50–4.75 times as long as Ant.VIb. On *Lathyrus japonicus* subsp. *japonicus* (Leguminosae). In Japan	*Megoura lathyricola* sp. n.
7	URS 0.89–1.17 times as long as 2HT and 0.68–0.88 times as long as Ant.VIb. Antennal tubercle weakly developed. Frons more than twice as wide as median depth. Antenna 0.83–0.94 times length of body. SIPH 1.07–1.31 times as long as Ant.III. On the genera *Lespedeza*, *Cajanus*, *Desmodium*, and *Indigofera* (Leguminosae). In Japan, China, India, Korea, Siberia, Switzerland, and Taiwan	*Megoura lespedezae*
–	URS 0.71–0.83 times as long as 2HT and 0.47–0.54 times as long as Ant.VIb. Antennal tubercle well developed. Frons V–shaped, as wide as median depth. Antenna at least 1.3 times length of body. SIPH shorter than Ant. III	8
8	Cauda about 0.50 times as long as SIPH. SIPH with narrow base, basal diameter shorter than middle diameter. Hairs on Ant.III about 0.50 times as long as BDAnt.III. On the genera *Indigofera*, *Hedysarum*, and *Tephrosia* (Leguminosae). In Afghanistan, India, Kashmir, Pakistan, and Thailand	*Megoura dooarsis*
–	Cauda more than 0.7 times as long as SIPH. SIPH widest at base. Hair on Ant.III 0.25 times as long as BDAnt.III. On the genus *Lespedeza* (Leguminosae). In Japan and Korea	*Megoura brevipilosa*

## Supplementary Material

XML Treatment for Megoura lathyricola
